# Effects of Sodium-Glucose Cotransporter-2 Inhibitors on Weight in Type 2 Diabetes Mellitus and Therapeutic Regimen Recommendation

**DOI:** 10.1155/2022/4491900

**Published:** 2022-03-18

**Authors:** Dong-Dong Wang, Yi-Zhen Mao, Yang Yang, Tian-Yun Wang, Ping Zhu, Su-Mei He, Xiao Chen

**Affiliations:** ^1^Jiangsu Key Laboratory of New Drug Research and Clinical Pharmacy & School of Pharmacy, Xuzhou Medical University, Xuzhou, Jiangsu 221004, China; ^2^School Infirmary, Jiangsu Normal University, Xuzhou, Jiangsu 221132, China; ^3^Department of Pharmacy, The Affiliated Changzhou Children's Hospital of Nantong University, Changzhou 213003, China; ^4^Department of Pharmacy, Huaian Hospital of Huaian City, Huaian, Jiangsu 223200, China; ^5^Department of Endocrinology, Huaian Hospital of Huaian City, Huaian, Jiangsu 223200, China; ^6^Department of Pharmacy, The Affiliated Suzhou Science & Technology Town Hospital of Nanjing Medical University, Suzhou Jiangsu 215153, China; ^7^Department of Pharmacy, Children's Hospital of Fudan University, Shanghai 201102, China

## Abstract

**Aims:**

The present study is aimed at exploring the effects of sodium-glucose cotransporter-2 (SGLT-2) inhibitors on weight in type 2 diabetes mellitus (T2DM) and therapeutic regimen recommendations.

**Methods:**

20,019 patients with T2DM were enrolled. The maximal effect (*E*_max_) models, whose evaluation index was change rate of body weight from baseline value, were used to analyze data using nonlinear mixed effect modeling (NONMEM).

**Results:**

For SGLT-2 inhibitors, canagliflozin, empagliflozin, ertugliflozin, ipragliflozin, luseogliflozin and tofogliflozin, the *E*_max_, and treatment duration to reach half of the maximal effects (ET_50_) were -3.72% and 3.35 weeks, -5.59% and 16.8 weeks, -2.84% and 3.42 weeks, -3.43% and 3.09 weeks, -3.04% and 4.38 weeks, and -2.45% and 3.16 weeks, respectively. In addition, for T2DM patients, 100 mg/day canagliflozin needs to be taken 13.4 weeks for the plateau of effect on weight; 10 mg/day empagliflozin needs to be taken 67.2 weeks for the plateau of effect on weight; 5 mg/day ertugliflozin needs to be taken 13.68 weeks for the plateau of effect on weight; 50 mg/day ipragliflozin needs to be taken 12.36 weeks for the plateau of effect on weight; 2.5 mg/day luseogliflozin needs to be taken 17.52 weeks for the plateau of effect on weight; 20 mg/day tofogliflozin needs to be taken 12.64 weeks for the plateau of effect on weight.

**Conclusions:**

This was the first study to explore effects of SGLT-2 inhibitors on weight in T2DM; meanwhile, the optimum dosages and treatment durations on weight from canagliflozin, empagliflozin, ertugliflozin, ipragliflozin, luseogliflozin, and tofogliflozin were recommended, respectively.

## 1. Introduction

The global epidemic trend of type 2 diabetes mellitus (T2DM) is becoming more and more serious, whose epidemiological data indicating that T2DM approximately impacts 1 in 11 adults [1]. Diabetes and its complications, such as diabetic angiocardiopathy, diabetic nephropathy, diabetic retinopathy, diabetic neuropathy, and diabetic hepatopathy, have serious impact on human health. Additional, T2DM patients are accompanied by dyslipidemia, atherosclerotic disease, hypertension, and obesity [2, 3], and what is serious is that more than 50% of T2DM patients have been reported with obesity [3, 4]. All we all know, T2DM patients with overweight or obesity are more likely to increase the risk of cardiovascular disease and lead to further risk increase of death, which are the important determinant of the prognosis of T2DM patients [4, 5]. Thus, it is vital to strengthen management of overweight or obesity in T2DM patients [6].

Sodium-glucose cotransporter-2 (SGLT-2) inhibitors, inhibiting SGLT-2 which is located in the S1 segment of renal proximal tubule and accounts for absorption of nearly 90% of glucose by kidney [7, 8], are a group of antidiabetic drugs. These drugs achieve their potential hypoglycemic activity by virtue of blocking the coupled reuptake of sodium and glucose in proximal tubule and promoting glycosuria [9]. In addition, apart from reducing blood glucose concentration, SGLT-2 inhibitors also have been demonstrated to have nonglycemic pleotropic effects, such as reducing risk of cardiovascular outcomes and mortality [10], attenuating hyperglycemia-induced vascular dysfunction [11], and inducting of weight loss, among which induction of weight loss is one of the important functions, whose mechanisms are due to osmotic diuresis and associated calorie losses [9, 12, 13]. However, the effects of SGLT-2 inhibitors on weight in T2DM are unclear; particularly, the dosages and treatment durations of SGLT-2 inhibitors lack clinical guidance. Therefore, the present study is aimed at exploring the effects of SGLT-2 inhibitors on weight in T2DM and therapeutic regimen recommendations.

## 2. Methods

### 2.1. Included Patients

T2DM patients treated with SGLT-2 inhibitors, including canagliflozin, empagliflozin, ertugliflozin, ipragliflozin, luseogliflozin, and tofogliflozin, were enrolled from published literatures, and the researches were approved by the ethics committee of each participating center [12, 14–69]. Search strategy was shown in Supplementary. The inclusion criteria were shown as follows: (a) T2DM patients; (b) with canagliflozin, empagliflozin, ertugliflozin, ipragliflozin, luseogliflozin, and tofogliflozin treatments; (c) randomized controlled trial (RCT); (d) with body weight information; and (e) exact doses and durations of canagliflozin, empagliflozin, ertugliflozin, ipragliflozin, luseogliflozin, and tofogliflozin. Source, grouping, common clinical dosages, duration of treatments, sample size, age, etc. were extracted from the above included studies. Studies identified for analysis were shown in Supplementary Table S1–S6, risk of bias was shown in Supplementary Figure S1–S6, and there was no obvious bias.

The change rates of body weight from baseline values were used as evaluation indices in order to eliminate the potential baseline effect, in which the formula (1) was as follows:

(1) $$\begin{array}{c} \text{EFF}\%=\frac{{\text{EFF}}_{\text{time}}-{\text{EFF}}_{\text{base}}}{{\text{EFF}}_{\text{base}}}\times 100\%. \end{array}$$

EFF_time_ is the value of weight at time, and EFF_base_ is the value of weight at baseline.

### 2.2. Model Establishment

The effects of canagliflozin, empagliflozin, ertugliflozin, ipragliflozin, luseogliflozin, and tofogliflozin on weight loss in T2DM patients were evaluated using the *E*_max_ models, respectively. Furthermore, the control effects should be subtracted from the sum effects for acquiring the actual effects on weight loss in T2DM from canagliflozin, empagliflozin, ertugliflozin, ipragliflozin, luseogliflozin, and tofogliflozin. The formulas (2) and (3) were as follows:

(2) $$\begin{array}{c} E_{c,k,i,j}=E_{a,k,i,j}–E_{b,k,i,j}, \end{array}$$

(3) $$\begin{array}{c} E_{c,k,i,j}=\frac{E_{\max,k,i,j}\times \text{Time}}{{\text{ET}}_{50,k,i,j}+\text{Time}}+\frac{{Ɛ}_{k,i,j}}{\sqrt{N_{k,i,j}/100}}. \end{array}$$

*E*_*a*,*k*,*i*,*j*_ was the sum effects on weight loss in T2DM patients; *E*_*b*,*k*,*i*,*j*_ was the control group effects on weight loss in T2DM patients; *E*_*c*,*k*,*i*,*j*_ was the actual effects on weight loss in T2DM patients; *k* represented SGLT-2 inhibitors, including canagliflozin, empagliflozin, ertugliflozin, ipragliflozin, luseogliflozin, and tofogliflozin; *i* was different studies; and *j* was time point of every study. *E*_max,*k*_ was the maximal effects on weight, ET_50,*k*_ was the treatment durations to reach half of the maximal effects on weight, Ɛ_*k*, *i*, *j*_ was the residual error of study *i* with *j* time under different SGLT-2 inhibitors, *N*_*k*,*i*,*j*_ was the sample size in study *i* with time point *j* under different SGLT-2 inhibitors, and Ɛ_*k*, *i*, *j*_ was weighted by sample size, assumed to be normally distributed, with a mean of 0 and variance of *σ*^2^/(*N*_*k*,*i*,*j*_/100).

The exponential error or additive error models were used to describe the variabilities of interstudies, in which the formulas (4)-(7) were as follows:

(4) $$\begin{array}{c} E_{\max,k,i,j}=E_{\max,k}\times \exp\left(\eta_{k,1,i}\right), \end{array}$$

(5) $$\begin{array}{c} \text{E}{\text{T}}_{50,k,i,j}=\text{E}{\text{T}}_{50,k}\times \exp\left(\eta_{k,2,i}\right), \end{array}$$

(6) $$\begin{array}{c} E_{\max,k,i,j}=E_{\max,k}+\eta_{k,1,i}, \end{array}$$

(7) $$\begin{array}{c} \text{E}{\text{T}}_{50,k,i,j}=\text{E}{\text{T}}_{50,k}+\eta_{k,2,i}. \end{array}$$

*η*_*k*,1,*i*_ and *η*_*k*,2,*i*_ were the interstudy variabilities, and when available, they would be added into *E*_max,*k*_ or ET_50, *k*_, respectively. *k* represented SGLT-2 inhibitors, including canagliflozin, empagliflozin, ertugliflozin, ipragliflozin, luseogliflozin, and tofogliflozin. *η*_*k*,1,*i*_ and *η*_*k*,2,*i*_ were assumed to normally distributed, with a mean of 0 and variance of *ω*_*k*,1,*i*_^2^ and *ω*_*k*,2,*i*_^2^, respectively.

In addition, continuous covariates and categorical covariates were evaluated by formulas (8)–(10):

(8) $$\begin{array}{c} P_{i}=P_{T}+\left(\text{COV}-\text{CO}{\text{V}}_{m}\right)\theta_{c}, \end{array}$$

(9) $$\begin{array}{c} P_{i}=P_{T}\times {\left(\text{COV}/{\text{COV}}_{m}\right)}^{\theta_{\text{c}}}, \end{array}$$

(10) $$\begin{array}{c} P_{i}=P_{T}+\text{COV}\times \theta_{c}. \end{array}$$

*P*_*i*_ was the parameter for a patient with a covariate value of COV, *P*_*T*_ was the typical value of the parameter, COV was covariate, and COV*_m_* was the median value of covariable in the population. *θ_c_* was a correction coefficient of the covariate to the model parameter.

The models were established using nonlinear mixed effect modeling (NONMEM, edition 7, ICON Development Solutions, Ellicott City, MD, USA) software. When the basic model was built up, potential covariates were considered for adding into *E*_max,*k*_ or ET_50, *k*_. The covariate inclusion criteria were change of objective function value (OFV), where the decrease of OFV was greater than 3.84 (*χ*^2^, *α* = 0.05, d.f. = 1), it was considered sufficient for inclusion. When the increase of OFV was greater than 6.63 (*χ*^2^, *α* = 0.01, d.f. = 1), it was considered sufficient for significance in the final model [70].

### 2.3. Model Validation

The individual predictions vs. observations and individual plots from SGLT-2 inhibitors, including canagliflozin, empagliflozin, ertugliflozin, ipragliflozin, luseogliflozin, and tofogliflozin, were used to estimate the final models, respectively. Prediction-corrected visual predictive check (VPC) plots were used to assess the predictive performance of final models.

### 2.4. Prediction

The curves of the final models from SGLT-2 inhibitors, including canagliflozin, empagliflozin, ertugliflozin, ipragliflozin, luseogliflozin, and tofogliflozin, were simulated using the Monte Carlo method, in addition, recommending the optimum dosages and treatment durations on weight in T2DM patients from canagliflozin, empagliflozin, ertugliflozin, ipragliflozin, luseogliflozin and tofogliflozin, respectively.

## 3. Results

### 3.1. Included Patients

A total of 20,019 patients with T2DM were enrolled in the present study, who were treated with SGLT-2 inhibitors, including canagliflozin, empagliflozin, ertugliflozin, ipragliflozin, luseogliflozin, and tofogliflozin, among which the dosages of canagliflozin, empagliflozin, ertugliflozin, ipragliflozin, luseogliflozin, and tofogliflozin were 100-300 mg/day, 10-25 mg/day, 5-15 mg/day, 50-100 mg/day, 2.5-5 mg/day, and 20-40 mg/day, respectively [12, 14–69].

### 3.2. Modeling

For canagliflozin, empagliflozin, ertugliflozin, ipragliflozin, luseogliflozin, and tofogliflozin, the *E*_max_ and ET_50_ were -3.72% and 3.35 weeks, -5.59% and 16.8 weeks, -2.84% and 3.42 weeks, -3.43% and 3.09 weeks, -3.04% and 4.38 weeks, and -2.45% and 3.16 weeks, respectively. The boostrap method results were shown in Supplementary Table S7, and estimate values were within the limits of 95% boostrap confidence interval. In these T2DM patients, no covariate (in particular dosage) was incorporated into models, showing no significant dosage response relationship within the current dose ranges. In other words, it was eligible to choose the lower dose of the dosage ranges, and for canagliflozin, empagliflozin, ertugliflozin, ipragliflozin, luseogliflozin, and tofogliflozin, the recommended dosages were 100 mg/day, 10 mg/day, 5 mg/day, 50 mg/day, 2.5 mg/day, and 20 mg/day, respectively.

In addition, the relationships between SGLT-2 inhibitors, including canagliflozin, empagliflozin, ertugliflozin, ipragliflozin, luseogliflozin and tofogliflozin, and loss of weight in T2DM patients, were shown in formulas (11)–(16), respectively:

(11) $$\begin{array}{c} \text{EFF}=\frac{-3.72\%\times \text{Time}}{3.35+\text{Time}}, \end{array}$$

(12) $$\begin{array}{c} \text{EFF}=\frac{-5.59\%\times \text{Time}}{16.8+\text{Time}}, \end{array}$$

(13) $$\begin{array}{c} \text{EFF}=\frac{-2.84\%\times \text{Time}}{3.42+\text{Time}}, \end{array}$$

(14) $$\begin{array}{c} \text{EFF}=\frac{-3.43\%\times \text{Time}}{3.09+\text{Time}}, \end{array}$$

(15) $$\begin{array}{c} \text{EFF}=\frac{-3.04\%\times \text{Time}}{4.38+\text{Time}}, \end{array}$$

(16) $$\begin{array}{c} \text{EFF}=\frac{-2.45\%\times \text{Time}}{3.16+\text{Time}}. \end{array}$$

EFF was canagliflozin, empagliflozin, ertugliflozin, ipragliflozin, luseogliflozin, and tofogliflozin on the effects of weight loss in T2DM patients. Time was canagliflozin, empagliflozin, ertugliflozin, ipragliflozin, luseogliflozin, and tofogliflozin treatment durations in T2DM patients.

### 3.3. Evaluation

**Figure 1:**
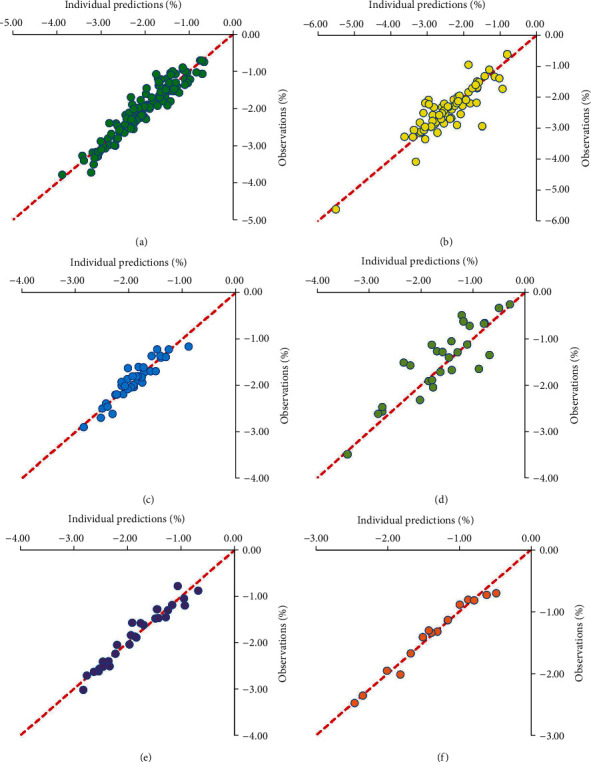
Visual inspection of routine diagnostic plots: (a) canagliflozin, (b) empagliflozin, (c) ertugliflozin, (d) ipragliflozin, (e) luseogliflozin, and (f) tofogliflozin.

**Figure 2:**
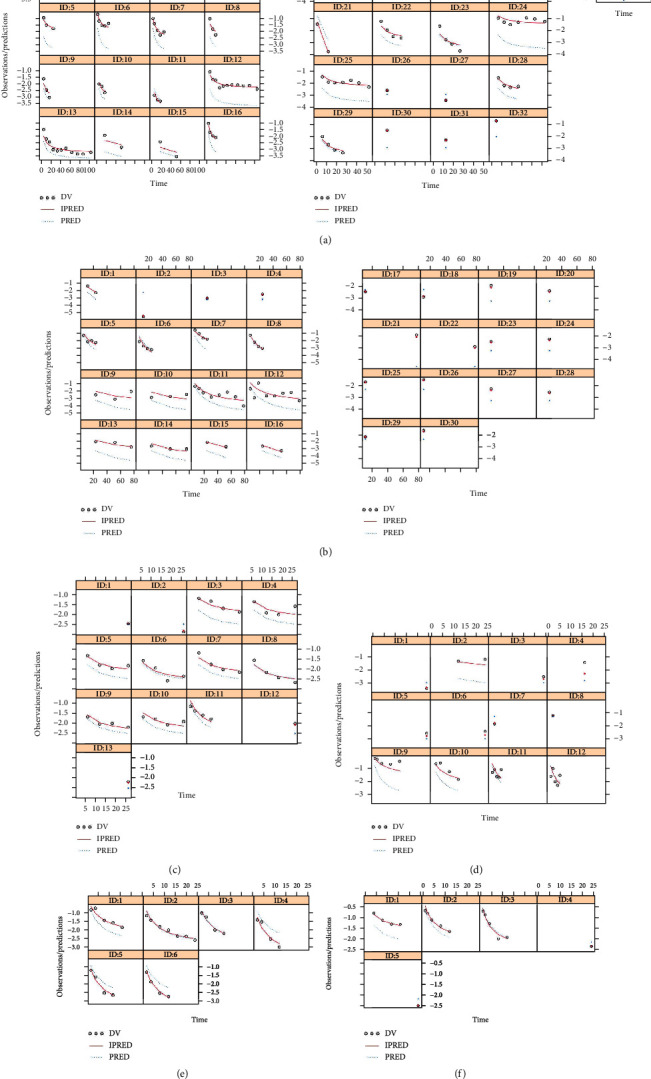
Individual plots: (a) canagliflozin, (b) empagliflozin, (c) ertugliflozin, (d) ipragliflozin, (e) luseogliflozin, and (f) tofogliflozin. Different IDs come from different groups of RCTs [12, 14–69].

**Figure 3:**
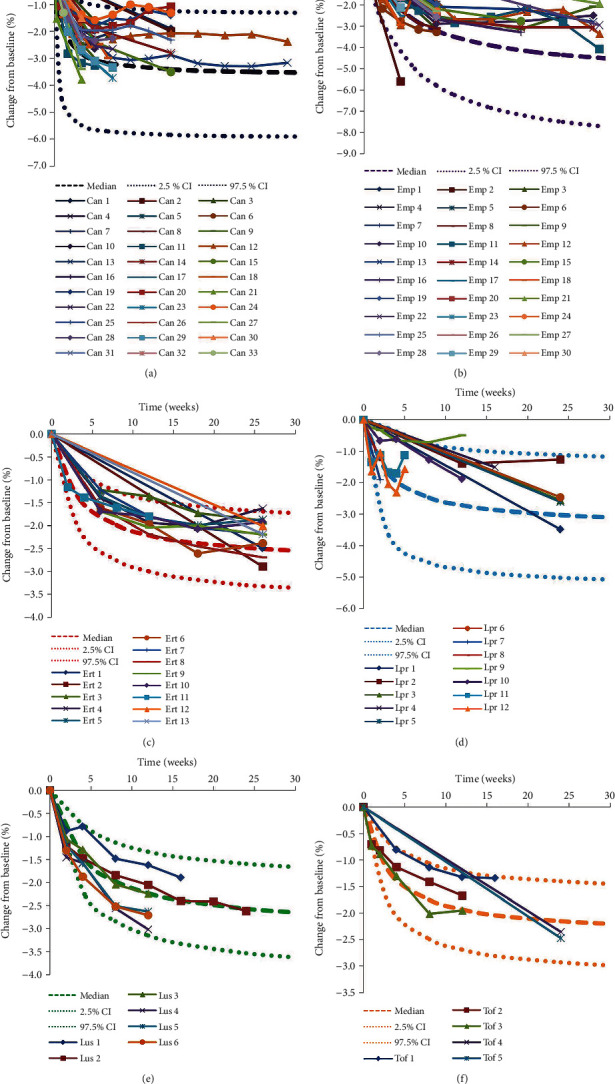
Prediction-corrected visual predictive check plots. (a) canagliflozin, (b) empagliflozin, (c) ertugliflozin, (d) ipragliflozin, (e) luseogliflozin, and (f) tofogliflozin. Median, 2.5% CI, and 97.5% CI were simulated by Monte Carlo (*n* = 1000); CI: confidence interval. Different color solid lines come from different groups of RCTs [12, 14–69].

The individual predictions vs. observations from canagliflozin, empagliflozin, ertugliflozin, ipragliflozin, luseogliflozin, and tofogliflozin models were shown in Figure 1, and Figures 1(a)–1(f) were from canagliflozin, empagliflozin, ertugliflozin, ipragliflozin, luseogliflozin, and tofogliflozin, respectively, showing good linear relationships between individual predictions and observations and indicating the better fitting of the final models. Individual plots were shown in Figure 2, and Figures 2(a)–2(f) were from canagliflozin, empagliflozin, ertugliflozin, ipragliflozin, luseogliflozin, and tofogliflozin, respectively, demonstrating acceptable predictability from the perspective of clinical sparse data. The prediction-corrected VPC plots were shown in Figure 3, and Figures 3(a)–3(f) were from canagliflozin, empagliflozin, ertugliflozin, ipragliflozin, luseogliflozin, and tofogliflozin, respectively, indicating that most observed data were included in the 95% prediction intervals produced with simulation data and meaning the predictive power of the final models.

### 3.4. Prediction

**Figure 4:**
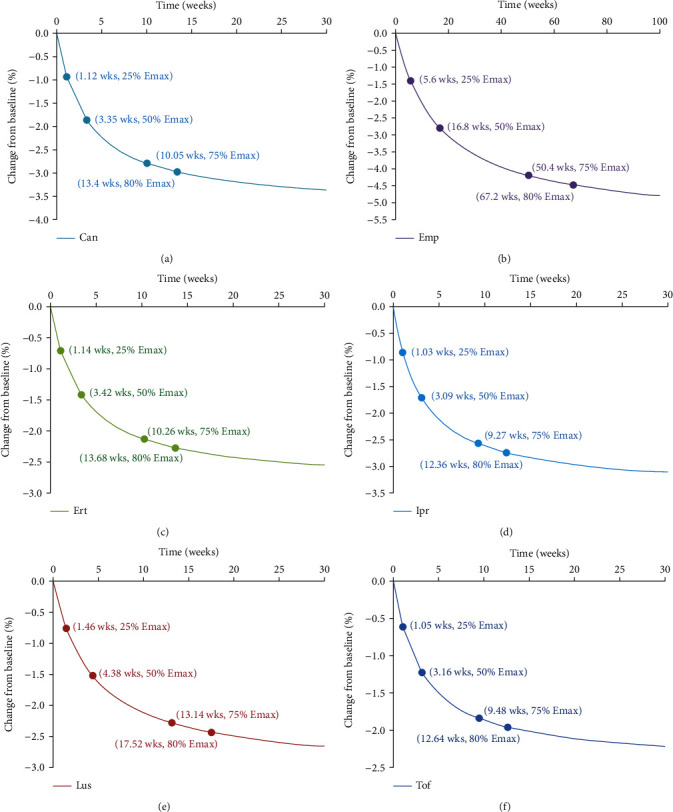
Model prediction: (a) canagliflozin, (b) empagliflozin, (c) ertugliflozin, (d) ipragliflozin, (e) luseogliflozin, and (f) tofogliflozin. wk: weeks.

The trends of efficacy of canagliflozin, empagliflozin, ertugliflozin, ipragliflozin, luseogliflozin, and tofogliflozin on the effects of weight loss in T2DM patients were shown in Figure 4. For canagliflozin, as shown in Figure 4(a), the duration to achieve 25%, 50%, 75%, and 80% of *E*_max_ was 1.12, 3.35, 10.05, and 13.4 weeks. For empagliflozin, as shown in Figure 4(b), the duration to achieve 25%, 50%, 75%, and 80% of *E*_max_ was 5.6, 16.8, 50.4, and 67.2 weeks. For ertugliflozin, as shown in Figure 4(c), the duration to achieve 25%, 50%, 75%, and 80% of *E*_max_ was 1.14, 3.42, 10.26, and 13.68 weeks. For ipragliflozin, as shown in Figure 4(d), the duration to achieve 25%, 50%, 75%, and 80% of *E*_max_ was 1.03, 3.09, 9.27, and 12.36 weeks. For luseogliflozin, as shown in Figure 4(e), the duration to achieve 25%, 50%, 75%, and 80% of *E*_max_ was 1.46, 4.38, 13.14, and 17.52 weeks. For tofogliflozin, as shown in Figure 4(f), the duration to achieve 25%, 50%, 75%, and 80% of *E*_max_ was 1.05, 3.16, 9.48, and 12.64 weeks.

In addition, as the study had found in the front section that the recommended dosages of canagliflozin, empagliflozin, ertugliflozin, ipragliflozin, luseogliflozin, and tofogliflozin were 100 mg/day, 10 mg/day, 5 mg/day, 50 mg/day, 2.5 mg/day, and 20 mg/day, respectively. Therefore, to achieve the plateau period (80% of *E*_max_) in loss of weight in T2DM patients, 100 mg/day canagliflozin needs to be taken 13.4 weeks for the plateau of effect on weight; 10 mg/day empagliflozin needs to be taken 67.2 weeks for the plateau of effect on weight; 5 mg/day ertugliflozin needs to be taken 13.68 weeks for the plateau of effect on weight; 50 mg/day ipragliflozin needs to be taken 12.36 weeks for the plateau of effect on weight; 2.5 mg/day luseogliflozin needs to be taken 17.52 weeks for the plateau of effect on weight; 20 mg/day tofogliflozin needs to be taken 12.64 weeks for the plateau of effect on weight.

## 4. Discussion

At present, many studies have found that SGLT-2 inhibitors, including canagliflozin, empagliflozin, ertugliflozin, ipragliflozin, luseogliflozin, and tofogliflozin, can reduce weight in T2DM patients, playing an important role in the treatment of T2DM [12, 14–69]. However, the effects of dosages and treatment durations of SGLT-2 inhibitors on weight in T2DM lack clinical guidance. Therefore, the present study is aimed at exploring the effects of SGLT-2 inhibitors on weight in T2DM and therapeutic regimen recommendations.

The present study adopts *E*_max_ models, the practical quantitative pharmacology tool, which can be used to explore the recommendation of drug dose and course of treatment in the course of disease treatment, and lay the foundation for the formulation of drug treatment plan. So far, many related studies have been reported. For example, Farhan et al. reported development and verification of a body weight-directed disease trial model for glucose homeostasis [71]. Chen et al. reported time course and dose effect of metformin on weight in patients with different disease states [72]. Li et al. reported comparative efficacy of nonhormonal drugs on menopausal hot flashes [73]. Wang et al. reported quantitative efficacy of L-carnitine supplementation on glycemic control in type 2 diabetes mellitus patients [74]. Chen et al. reported analysis of time course and dose effect of tacrolimus on proteinuria in lupus nephritis patients [75]. Li et al. reported quantitative efficacy of soy isoflavones on menopausal hot flashes [76]. Thus, we used this utility tool to explore the optimum dosages and treatment durations on weight from canagliflozin, empagliflozin, ertugliflozin, ipragliflozin, luseogliflozin, and tofogliflozin, respectively.

The nonlinear mixed effect modeling (NONMEM) was used to analyze. In the process of our research, the evaluation index was change rate of body weight from baseline value in order to eliminate the potential baseline effect. In addition, the control effects were subtracted from the sum effects for acquiring the actual effects on weight loss in T2DM from canagliflozin, empagliflozin, ertugliflozin, ipragliflozin, luseogliflozin, and tofogliflozin. Finally, for canagliflozin, empagliflozin, ertugliflozin, ipragliflozin, luseogliflozin, and tofogliflozin, the *E*_max_ and ET_50_ were -3.72% and 3.35 weeks, -5.59% and 16.8 weeks, -2.84% and 3.42 weeks, -3.43% and 3.09 weeks, -3.04% and 4.38 weeks, and -2.45% and 3.16 weeks, respectively. The order of efficacy of canagliflozin, empagliflozin, ertugliflozin, ipragliflozin, luseogliflozin, and tofogliflozin on the effects of weight loss in T2DM patients from large to small was 10 mg/day empagliflozin, 100 mg/day canagliflozin, 50 mg/day ipragliflozin, 2.5 mg/day luseogliflozin, 5 mg/day ertugliflozin, and 20 mg/day tofogliflozin. The onset time of weight loss from fast to slow was 50 mg/day ipragliflozin, 20 mg/day tofogliflozin, 100 mg/day canagliflozin, 5 mg/day ertugliflozin, 2.5 mg/day luseogliflozin, and 10 mg/day empagliflozin.

Besides, the optimum dosages and treatment durations on weight from canagliflozin, empagliflozin, ertugliflozin, ipragliflozin, luseogliflozin, and tofogliflozin were recommended in T2DM patients, respectively. 100 mg/day canagliflozin needs to be taken 13.4 weeks for the plateau of effect on weight; 10 mg/day empagliflozin needs to be taken 67.2 weeks for the plateau of effect on weight; 5 mg/day ertugliflozin needs to be taken 13.68 weeks for the plateau of effect on weight; 50 mg/day ipragliflozin needs to be taken 12.36 weeks for the plateau of effect on weight; 2.5 mg/day luseogliflozin needs to be taken 17.52 weeks for the plateau of effect on weight; 20 mg/day tofogliflozin needs to be taken 12.64 weeks for the plateau of effect on weight.

The present study firstly explored the effects of canagliflozin, empagliflozin, ertugliflozin, ipragliflozin, luseogliflozin, and tofogliflozin on weight in T2DM and recommended therapeutic regimen. However, this study also had some limitations. For example, the studies of luseogliflozin and tofogliflozin were all from Japan and lack of data on other countries' populations. This required further population expansion and inclusion of populations from more countries in future studies.

## 5. Conclusion

This was the first comprehensive study to explore effects of SGLT-2 inhibitors on weight in T2DM; meanwhile, the optimum dosages and treatment durations on weight from canagliflozin, empagliflozin, ertugliflozin, ipragliflozin, luseogliflozin, and tofogliflozin were recommended, respectively.

## Data Availability

The data related to this article can be publicly available after the article accepted.

## Supplementary Materials

Supplementary Materials The Supplementary Materials have been submitted along with the primary manuscript, including search strategy, studies identified for analysis, risk of bias, and parameter estimates of final models and boostrap.
