# Supplementary material for: Effects of Sodium-Glucose Cotransporter-2 Inhibitors on Weight in Type 2 Diabetes Mellitus and Therapeutic Regimen Recommendation
Source: J Diabetes Res. 2022 Mar 18;2022:4491900. doi: 10.1155/2022/4491900 (PMC8956429; doi:10.1155/2022/4491900)
Supplement: Supplementary Materials — The Supplementary Materials have been submitted along with the primary manuscript, including search strategy, studies identified for analysis, risk of bias, and parameter estimates of final models and boostrap. [file 4491900.f1.docx]

**Supplementary**

**Search strategy in Pubmed (**[**https://pubmed.ncbi.nlm.nih.gov/**](https://pubmed.ncbi.nlm.nih.gov/)**)**

**1. Canagliflozin**

1.1. Search strategy

(((canagliflozin[Title]) OR (Invokana[Title])) AND (((((type 2 diabetes[Title]) OR (type 2 diabetes mellitus[Title])) OR (T2D[Title])) OR (T2DM[Title])) OR (type 2 diabetic patients[Title]))) AND ((((weight) OR (bodyweight)) OR (body mass index)) OR (BMI))

1.2. List of final included studies (n=18)

1: Yale JF, Xie J, Sherman SE, Garceau C. Canagliflozin in Conjunction With Sulfonylurea Maintains Glycemic Control and Weight Loss Over 52 Weeks: A Randomized, Controlled Trial in Patients With Type 2 Diabetes Mellitus. Clin Ther. 2017 Nov;39(11):2230-2242.e2. doi: 10.1016/j.clinthera.2017.10.003. Epub 2017 Nov 3. PMID: 29103664.

2: Kadowaki T, Inagaki N, Kondo K, Nishimura K, Kaneko G, Maruyama N, Nakanishi N, Iijima H, Watanabe Y, Gouda M. Efficacy and safety of canagliflozin as add-on therapy to teneligliptin in Japanese patients with type 2 diabetes mellitus: Results of a 24-week, randomized, double-blind, placebo-controlled trial. Diabetes Obes Metab. 2017 Jun;19(6):874-882. doi: 10.1111/dom.12898. Epub 2017 Mar 31. PMID: 28177187; PMCID: PMC5484989.

3: Inagaki N, Harashima S, Maruyama N, Kawaguchi Y, Goda M, Iijima H. Efficacy and safety of canagliflozin in combination with insulin: a double-blind, randomized, placebo-controlled study in Japanese patients with type 2 diabetes mellitus. Cardiovasc Diabetol. 2016 Jun 18;15:89. doi: 10.1186/s12933-016-0407-4. PMID: 27316668; PMCID: PMC4912792.

4: Rodbard HW, Seufert J, Aggarwal N, Cao A, Fung A, Pfeifer M, Alba M. Efficacy and safety of titrated canagliflozin in patients with type 2 diabetes mellitus inadequately controlled on metformin and sitagliptin. Diabetes Obes Metab. 2016 Aug;18(8):812-9. doi: 10.1111/dom.12684. Epub 2016 Jun 7. PMID: 27160639; PMCID: PMC5089595.

5: Rosenstock J, Chuck L, González-Ortiz M, Merton K, Craig J, Capuano G, Qiu R. Initial Combination Therapy With Canagliflozin Plus Metformin Versus Each Component as Monotherapy for Drug-Naïve Type 2 Diabetes. Diabetes Care. 2016 Mar;39(3):353-62. doi: 10.2337/dc15-1736. Epub 2016 Jan 19. PMID: 26786577.

6: Fulcher G, Matthews DR, Perkovic V, de Zeeuw D, Mahaffey KW, Mathieu C, Woo V, Wysham C, Capuano G, Desai M, Shaw W, Vercruysse F, Meininger G, Neal B; CANVAS trial collaborative group. Efficacy and safety of canagliflozin when used in conjunction with incretin-mimetic therapy in patients with type 2 diabetes. Diabetes Obes Metab. 2016 Jan;18(1):82-91. doi: 10.1111/dom.12589. Epub 2015 Dec 8. PMID: 26450639.

7: Bode B, Stenlöf K, Harris S, Sullivan D, Fung A, Usiskin K, Meininger G. Long-term efficacy and safety of canagliflozin over 104 weeks in patients aged 55-80 years with type 2 diabetes. Diabetes Obes Metab. 2015 Mar;17(3):294-303. doi: 10.1111/dom.12428. Epub 2015 Jan 12. PMID: 25495720.

8: Neal B, Perkovic V, de Zeeuw D, Mahaffey KW, Fulcher G, Ways K, Desai M, Shaw W, Capuano G, Alba M, Jiang J, Vercruysse F, Meininger G, Matthews D; CANVAS Trial Collaborative Group. Efficacy and safety of canagliflozin, an inhibitor of sodium-glucose cotransporter 2, when used in conjunction with insulin therapy in patients with type 2 diabetes. Diabetes Care. 2015 Mar;38(3):403-11. doi: 10.2337/dc14-1237. Epub 2014 Dec 2. PMID: 25468945.

9: Ji L, Han P, Liu Y, Yang G, Dieu Van NK, Vijapurkar U, Qiu R, Meininger G. Canagliflozin in Asian patients with type 2 diabetes on metformin alone or metformin in combination with sulphonylurea. Diabetes Obes Metab. 2015 Jan;17(1):23-31. doi: 10.1111/dom.12385. Epub 2014 Oct 14. PMID: 25175734.

10: Inagaki N, Kondo K, Yoshinari T, Takahashi N, Susuta Y, Kuki H. Efficacy and safety of canagliflozin monotherapy in Japanese patients with type 2 diabetes inadequately controlled with diet and exercise: a 24-week, randomized, double-blind, placebo-controlled, Phase III study. Expert Opin Pharmacother. 2014 Aug;15(11):1501-15. doi: 10.1517/14656566.2014.935764. PMID: 25010793.

11: Yale JF, Bakris G, Cariou B, Nieto J, David-Neto E, Yue D, Wajs E, Figueroa K, Jiang J, Law G, Usiskin K, Meininger G; DIA3004 Study Group. Efficacy and safety of canagliflozin over 52 weeks in patients with type 2 diabetes mellitus and chronic kidney disease. Diabetes Obes Metab. 2014 Oct;16(10):1016-27. doi: 10.1111/dom.12348. Epub 2014 Jul 22. PMID: 24965700.

12: Sha S, Polidori D, Heise T, Natarajan J, Farrell K, Wang SS, Sica D, Rothenberg P, Plum-Mörschel L. Effect of the sodium glucose co-transporter 2 inhibitor canagliflozin on plasma volume in patients with type 2 diabetes mellitus. Diabetes Obes Metab. 2014 Nov;16(11):1087-95. doi: 10.1111/dom.12322. Epub 2014 Jul 8. PMID: 24939043.

13: Forst T, Guthrie R, Goldenberg R, Yee J, Vijapurkar U, Meininger G, Stein P. Efficacy and safety of canagliflozin over 52 weeks in patients with type 2 diabetes on background metformin and pioglitazone. Diabetes Obes Metab. 2014 May;16(5):467-77. doi: 10.1111/dom.12273. Epub 2014 Mar 12. PMID: 24528605; PMCID: PMC4237547.

14: Wilding JP, Charpentier G, Hollander P, González-Gálvez G, Mathieu C, Vercruysse F, Usiskin K, Law G, Black S, Canovatchel W, Meininger G. Efficacy and safety of canagliflozin in patients with type 2 diabetes mellitus inadequately controlled with metformin and sulphonylurea: a randomised trial. Int J Clin Pract. 2013 Dec;67(12):1267-82. doi: 10.1111/ijcp.12322. Epub 2013

Oct 13. PMID: 24118688; PMCID: PMC4282288.

15: Inagaki N, Kondo K, Yoshinari T, Maruyama N, Susuta Y, Kuki H. Efficacy and safety of canagliflozin in Japanese patients with type 2 diabetes: a randomized, double-blind, placebo-controlled, 12-week study. Diabetes Obes Metab. 2013 Dec;15(12):1136-45. doi: 10.1111/dom.12149. Epub 2013 Jul 14. PMID: 23782594; PMCID: PMC3906835.

16: Stenlöf K, Cefalu WT, Kim KA, Alba M, Usiskin K, Tong C, Canovatchel W, Meininger G. Efficacy and safety of canagliflozin monotherapy in subjects with type 2 diabetes mellitus inadequately controlled with diet and exercise. Diabetes Obes Metab. 2013 Apr;15(4):372-82. doi: 10.1111/dom.12054. Epub 2013 Jan 24. PMID: 23279307; PMCID: PMC3593184.

17: Rosenstock J, Aggarwal N, Polidori D, Zhao Y, Arbit D, Usiskin K, Capuano G, Canovatchel W; Canagliflozin DIA 2001 Study Group. Dose-ranging effects of canagliflozin, a sodium-glucose cotransporter 2 inhibitor, as add-on to metformin in subjects with type 2 diabetes. Diabetes Care. 2012 Jun;35(6):1232-8. doi: 10.2337/dc11-1926. Epub 2012 Apr 9. PMID: 22492586; PMCID: PMC3357223.

18: Devineni D, Morrow L, Hompesch M, Skee D, Vandebosch A, Murphy J, Ways K, Schwartz S. Canagliflozin improves glycaemic control over 28 days in subjects with type 2 diabetes not optimally controlled on insulin. Diabetes Obes Metab. 2012 Jun;14(6):539-45. doi: 10.1111/j.1463-1326.2012.01558.x. Epub 2012 Feb 8. PMID: 22226086.

**2. Empagliflozin**

2.1. Search strategy

(((empagliflozin[Title]) OR (Jardiance[Title])) AND (((((type 2 diabetes[Title]) OR (type 2 diabetes mellitus[Title])) OR (T2D[Title])) OR (T2DM[Title])) OR (type 2 diabetic patients[Title]))) AND ((((weight) OR (bodyweight)) OR (body mass index)) OR (BMI))

2.2. List of final included studies (n=15)

1: Kahl S, Gancheva S, Straßburger K, Herder C, Machann J, Katsuyama H, Kabisch S, Henkel E, Kopf S, Lagerpusch M, Kantartzis K, Kupriyanova Y, Markgraf D, van Gemert T, Knebel B, Wolkersdorfer MF, Kuss O, Hwang JH, Bornstein SR, Kasperk C, Stefan N, Pfeiffer A, Birkenfeld AL, Roden M. Empagliflozin Effectively Lowers Liver Fat Content in Well-Controlled Type 2 Diabetes: A Randomized, Double-Blind, Phase 4, Placebo-Controlled Trial. Diabetes Care. 2020 Feb;43(2):298-305. doi: 10.2337/dc19-0641. Epub 2019 Sep 20. PMID: 31540903.

2: Hattori S. Empagliflozin decreases remnant-like particle cholesterol in type 2 diabetes patients with insulin resistance. J Diabetes Investig. 2018 Jul;9(4):870-874. doi: 10.1111/jdi.12781. Epub 2017 Dec 27. PMID: 29193767; PMCID: PMC6031503.

3: Søfteland E, Meier JJ, Vangen B, Toorawa R, Maldonado-Lutomirsky M, Broedl UC. Empagliflozin as Add-on Therapy in Patients With Type 2 Diabetes Inadequately Controlled With Linagliptin and Metformin: A 24-Week Randomized, Double-Blind, Parallel-Group Trial. Diabetes Care. 2017 Feb;40(2):201-209. doi: 10.2337/dc16-1347. Epub 2016 Dec 2. PMID: 27913576.

4: Hadjadj S, Rosenstock J, Meinicke T, Woerle HJ, Broedl UC. Initial Combination of Empagliflozin and Metformin in Patients With Type 2 Diabetes. Diabetes Care. 2016 Oct;39(10):1718-28. doi: 10.2337/dc16-0522. Epub 2016 Aug 4. PMID: 27493136.

5: Roden M, Merker L, Christiansen AV, Roux F, Salsali A, Kim G, Stella P, Woerle HJ, Broedl UC; EMPA-REG EXTEND™ MONO investigators. Safety, tolerability and effects on cardiometabolic risk factors of empagliflozin monotherapy in drug-naïve patients with type 2 diabetes: a double-blind extension of a Phase III randomized controlled trial. Cardiovasc Diabetol. 2015 Dec 23;14:154. doi: 10.1186/s12933-015-0314-0. PMID: 26701110; PMCID: PMC4690334.

6: Rosenstock J, Jelaska A, Zeller C, Kim G, Broedl UC, Woerle HJ; EMPA-REG BASALTM trial investigators. Impact of empagliflozin added on to basal insulin in type 2 diabetes inadequately controlled on basal insulin: a 78-week randomized, double-blind, placebo-controlled trial. Diabetes Obes Metab. 2015 Oct;17(10):936-48. doi: 10.1111/dom.12503. Epub 2015 Jul 14. PMID: 26040302; PMCID: PMC5034797.

7: Merker L, Häring HU, Christiansen AV, Roux F, Salsali A, Kim G, Meinicke T, Woerle HJ, Broedl UC; EMPA-REG EXTEND MET investigators. Empagliflozin as add-on to metformin in people with Type 2 diabetes. Diabet Med. 2015 Dec;32(12):1555-67. doi: 10.1111/dme.12814. Epub 2015 Jul 14. PMID: 26031566.

8: DeFronzo RA, Lewin A, Patel S, Liu D, Kaste R, Woerle HJ, Broedl UC. Combination of empagliflozin and linagliptin as second-line therapy in subjects with type 2 diabetes inadequately controlled on metformin. Diabetes Care. 2015 Mar;38(3):384-93. doi: 10.2337/dc14-2364. Epub 2015 Jan 12. Erratum in: Diabetes Care. 2015 Jun;38(6):1173. PMID: 25583754.

9: Kadowaki T, Haneda M, Inagaki N, Terauchi Y, Taniguchi A, Koiwai K, Rattunde H, Woerle HJ, Broedl UC. Empagliflozin monotherapy in Japanese patients with type 2 diabetes mellitus: a randomized, 12-week, double-blind, placebo-controlled, phase II trial. Adv Ther. 2014 Jun;31(6):621-38. doi: 10.1007/s12325-014-0126-8. Epub 2014 Jun 24. PMID: 24958326.

10: Häring HU, Merker L, Seewaldt-Becker E, Weimer M, Meinicke T, Broedl UC, Woerle HJ; EMPA-REG MET Trial Investigators. Empagliflozin as add-on to metformin in patients with type 2 diabetes: a 24-week, randomized, double-blind, placebo-controlled trial. Diabetes Care. 2014 Jun;37(6):1650-9. doi: 10.2337/dc13-2105. Epub 2014 Apr 10. PMID: 24722494.

11: Ferrannini E, Berk A, Hantel S, Pinnetti S, Hach T, Woerle HJ, Broedl UC. Long-term safety and efficacy of empagliflozin, sitagliptin, and metformin: an active-controlled, parallel-group, randomized, 78-week open-label extension study in patients with type 2 diabetes. Diabetes Care. 2013 Dec;36(12):4015-21. doi: 10.2337/dc13-0663. Epub 2013 Nov 1. PMID: 24186878; PMCID: PMC3836134.

12: Kovacs CS, Seshiah V, Swallow R, Jones R, Rattunde H, Woerle HJ, Broedl UC; EMPA-REG PIO™ trial investigators. Empagliflozin improves glycaemic and weight control as add-on therapy to pioglitazone or pioglitazone plus metformin in patients with type 2 diabetes: a 24-week, randomized, placebo-controlled trial. Diabetes Obes Metab. 2014 Feb;16(2):147-58. doi: 10.1111/dom.12188. Epub 2013 Aug 22. PMID: 23906415.

13: Rosenstock J, Seman LJ, Jelaska A, Hantel S, Pinnetti S, Hach T, Woerle HJ. Efficacy and safety of empagliflozin, a sodium glucose cotransporter 2 (SGLT2) inhibitor, as add-on to metformin in type 2 diabetes with mild hyperglycaemia. Diabetes Obes Metab. 2013 Dec;15(12):1154-60. doi: 10.1111/dom.12185. Epub 2013 Aug 22. PMID: 23906374.

14: Häring HU, Merker L, Seewaldt-Becker E, Weimer M, Meinicke T, Woerle HJ, Broedl UC; EMPA-REG METSU Trial Investigators. Empagliflozin as add-on to metformin plus sulfonylurea in patients with type 2 diabetes: a 24-week, randomized, double-blind, placebo-controlled trial. Diabetes Care. 2013 Nov;36(11):3396-404. doi: 10.2337/dc12-2673. Epub 2013 Aug 20. PMID: 23963895; PMCID: PMC3816918.

15: Ferrannini E, Seman L, Seewaldt-Becker E, Hantel S, Pinnetti S, Woerle HJ. A Phase IIb, randomized, placebo-controlled study of the SGLT2 inhibitor empagliflozin in patients with type 2 diabetes. Diabetes Obes Metab. 2013 Aug;15(8):721-8. doi: 10.1111/dom.12081. Epub 2013 Mar 4. PMID: 23398530.

**3. Ertugliflozin**

3.1. Search strategy

((ertugliflozin[Title]) AND (((((type 2 diabetes[Title]) OR (type 2 diabetes mellitus[Title])) OR (T2D[Title])) OR (T2DM[Title])) OR (type 2 diabetic patients[Title]))) AND ((((weight) OR (bodyweight)) OR (body mass index)) OR (BMI))

3.2. List of final included studies (n=7)

1: Ji L, Liu Y, Miao H, Xie Y, Yang M, Wang W, Mu Y, Yan P, Pan S, Lauring B, Liu S, Huyck S, Qiu Y, Terra SG. Safety and efficacy of ertugliflozin in Asian patients with type 2 diabetes mellitus inadequately controlled with metformin monotherapy: VERTIS Asia. Diabetes Obes Metab. 2019 Jun;21(6):1474-1482. doi: 10.1111/dom.13681. Epub 2019 Apr 5. PMID: 30830724; PMCID: PMC7379575.

2: Gallo S, Charbonnel B, Goldman A, Shi H, Huyck S, Darekar A, Lauring B, Terra SG. Long-term efficacy and safety of ertugliflozin in patients with type 2 diabetes mellitus inadequately controlled with metformin monotherapy: 104-week VERTIS MET trial. Diabetes Obes Metab. 2019 Apr;21(4):1027-1036. doi: 10.1111/dom.13631. Epub 2019 Feb 14. PMID: 30614616; PMCID: PMC6593724.

3: Aronson R, Frias J, Goldman A, Darekar A, Lauring B, Terra SG. Long-term efficacy and safety of ertugliflozin monotherapy in patients with inadequately controlled T2DM despite diet and exercise: VERTIS MONO extension study. Diabetes Obes Metab. 2018 Jun;20(6):1453-1460. doi: 10.1111/dom.13251. Epub 2018 Feb 23. PMID: 29419917; PMCID: PMC5969239.

4: Pratley RE, Eldor R, Raji A, Golm G, Huyck SB, Qiu Y, Sunga S, Johnson J, Terra SG, Mancuso JP, Engel SS, Lauring B. Ertugliflozin plus sitagliptin versus either individual agent over 52 weeks in patients with type 2 diabetes mellitus inadequately controlled with metformin: The VERTIS FACTORIAL randomized trial. Diabetes Obes Metab. 2018 May;20(5):1111-1120. doi: 10.1111/dom.13194. Epub 2018 Jan 25. PMID: 29266675; PMCID: PMC5947297.

5: Dagogo-Jack S, Liu J, Eldor R, Amorin G, Johnson J, Hille D, Liao Y, Huyck S, Golm G, Terra SG, Mancuso JP, Engel SS, Lauring B. Efficacy and safety of the addition of ertugliflozin in patients with type 2 diabetes mellitus inadequately controlled with metformin and sitagliptin: The VERTIS SITA2 placebo-controlled randomized study. Diabetes Obes Metab. 2018 Mar;20(3):530-540. doi: 10.1111/dom.13116. Epub 2017 Oct 23. PMID: 28921862; PMCID: PMC5836931.

6: Amin NB, Wang X, Jain SM, Lee DS, Nucci G, Rusnak JM. Dose-ranging efficacy and safety study of ertugliflozin, a sodium-glucose co-transporter 2 inhibitor, in patients with type 2 diabetes on a background of metformin. Diabetes Obes Metab. 2015 Jun;17(6):591-598. doi: 10.1111/dom.12460. Epub 2015 Mar 31. PMID: 25754396.

7: Grunberger G, Camp S, Johnson J, Huyck S, Terra SG, Mancuso JP, Jiang ZW, Golm G, Engel SS, Lauring B. Ertugliflozin in Patients with Stage 3 Chronic Kidney Disease and Type 2 Diabetes Mellitus: The VERTIS RENAL Randomized Study. Diabetes Ther. 2018 Feb;9(1):49-66. doi: 10.1007/s13300-017-0337-5. Epub 2017 Nov 20. PMID: 29159457; PMCID: PMC5801223.

**4. Ipragliflozin**

4.1. Search strategy

(((ipragliflozin[Title]) OR (Suglat[Title])) AND (((((type 2 diabetes[Title]) OR (type 2 diabetes mellitus[Title])) OR (T2D[Title])) OR (T2DM[Title])) OR (type 2 diabetic patients[Title]))) AND ((((weight) OR (bodyweight)) OR (body mass index)) OR (BMI))

4.2. List of final included studies (n=10)

1: Inoue H, Morino K, Ugi S, Tanaka-Mizuno S, Fuse K, Miyazawa I, Kondo K, Sato D, Ohashi N, Ida S, Sekine O, Yoshimura M, Murata K, Miura K, Arima H, Maegawa H; SUMS-ADDIT-1 Research group. Ipragliflozin, a sodium-glucose cotransporter 2 inhibitor, reduces bodyweight and fat mass, but not muscle mass, in Japanese type 2 diabetes patients treated with insulin: A randomized clinical trial. J Diabetes Investig. 2019 Jul;10(4):1012-1021. doi: 10.1111/jdi.12985. Epub 2019 Jan 21. PMID: 30536746; PMCID: PMC6626939.

2: Shestakova MV, Wilding JPH, Wilpshaar W, Tretter R, Orlova VL, Verbovoy AF. A phase 3 randomized placebo-controlled trial to assess the efficacy and safety of ipragliflozin as an add-on therapy to metformin in Russian patients with inadequately controlled type 2 diabetes mellitus. Diabetes Res Clin Pract. 2018 Dec;146:240-250. doi: 10.1016/j.diabres.2018.10.018. Epub 2018 Nov 1. PMID:

30391333.

3: Han KA, Chon S, Chung CH, Lim S, Lee KW, Baik S, Jung CH, Kim DS, Park KS, Yoon KH, Lee IK, Cha BS, Sakatani T, Park S, Lee MK. Efficacy and safety of ipragliflozin as an add-on therapy to sitagliptin and metformin in Korean patients with inadequately controlled type 2 diabetes mellitus: A randomized controlled trial. Diabetes Obes Metab. 2018 Oct;20(10):2408-2415. doi: 10.1111/dom.13394. Epub 2018 Jul 16. PMID: 29862619; PMCID: PMC6175352.

4: Ishihara H, Yamaguchi S, Nakao I, Okitsu A, Asahina S. Efficacy and safety of ipragliflozin as add-on therapy to insulin in Japanese patients with type 2 diabetes mellitus (IOLITE): a multi-centre, randomized, placebo-controlled, double-blind study. Diabetes Obes Metab. 2016 Dec;18(12):1207-1216. doi: 10.1111/dom.12745. Epub 2016 Sep 15. PMID: 27436788; PMCID: PMC5484364.

5: Kashiwagi A, Takahashi H, Ishikawa H, Yoshida S, Kazuta K, Utsuno A, Ueyama E. A randomized, double-blind, placebo-controlled study on long-term efficacy and safety of ipragliflozin treatment in patients with type 2 diabetes mellitus and renal impairment: results of the long-term ASP1941 safety evaluation in patients with type 2 diabetes with renal impairment (LANTERN) study. Diabetes Obes Metab. 2015 Feb;17(2):152-60. doi: 10.1111/dom.12403. PMID: 25347938; PMCID: PMC5024052.

6: Kashiwagi A, Kazuta K, Goto K, Yoshida S, Ueyama E, Utsuno A. Ipragliflozin in combination with metformin for the treatment of Japanese patients with type 2 diabetes: ILLUMINATE, a randomized, double-blind, placebo-controlled study. Diabetes Obes Metab. 2015 Mar;17(3):304-8. doi: 10.1111/dom.12331. Epub 2014 Jul 31. PMID: 24919820; PMCID: PMC4342773.

7: Kadokura T, Akiyama N, Kashiwagi A, Utsuno A, Kazuta K, Yoshida S, Nagase I, Smulders R, Kageyama S. Pharmacokinetic and pharmacodynamic study of ipragliflozin in Japanese patients with type 2 diabetes mellitus: a randomized, double-blind, placebo-controlled study. Diabetes Res Clin Pract. 2014 Oct;106(1):50-6. doi: 10.1016/j.diabres.2014.07.020. Epub 2014 Jul 26. PMID: 25149596.

8: Fonseca VA, Ferrannini E, Wilding JP, Wilpshaar W, Dhanjal P, Ball G, Klasen S. Active- and placebo-controlled dose-finding study to assess the efficacy, safety, and tolerability of multiple doses of ipragliflozin in patients with type 2 diabetes mellitus. J Diabetes Complications. 2013 May-Jun;27(3):268-73. doi: 10.1016/j.jdiacomp.2012.11.005. Epub 2012 Dec 29. PMID: 23276620.

9: Wilding JP, Ferrannini E, Fonseca VA, Wilpshaar W, Dhanjal P, Houzer A. Efficacy and safety of ipragliflozin in patients with type 2 diabetes inadequately controlled on metformin: a dose-finding study. Diabetes Obes Metab. 2013 May;15(5):403-9. doi: 10.1111/dom.12038. Epub 2012 Dec 7. PMID: 23163880.

10: Schwartz SL, Akinlade B, Klasen S, Kowalski D, Zhang W, Wilpshaar W. Safety, pharmacokinetic, and pharmacodynamic profiles of ipragliflozin (ASP1941), a novel and selective inhibitor of sodium-dependent glucose co-transporter 2, in patients with type 2 diabetes mellitus. Diabetes Technol Ther. 2011 Dec;13(12):1219-27. doi: 10.1089/dia.2011.0012. Epub 2011 Aug 19. PMID: 21854192.

**5. Luseogliflozin**

5.1. Search strategy

(((luseogliflozin[Title]) OR (Lusefi[Title])) AND (((((type 2 diabetes[Title]) OR (type 2 diabetes mellitus[Title])) OR (T2D[Title])) OR (T2DM[Title])) OR (type 2 diabetic patients[Title]))) AND ((((weight) OR (bodyweight)) OR (body mass index)) OR (BMI))

5.2. List of final included studies (n=4)

1: Seino Y, Sasaki T, Fukatsu A, Imazeki H, Ochiai H, Sakai S. Efficacy and safety of luseogliflozin added to insulin therapy in Japanese patients with type 2 diabetes: a multicenter, 52-week, clinical study with a 16-week, double-blind period and a 36-week, open-label period. Curr Med Res Opin. 2018 Jun;34(6):981-994. doi: 10.1080/03007995.2018.1441816. Epub 2018 Mar 20. PMID: 29448833.

2: Seino Y, Sasaki T, Fukatsu A, Ubukata M, Sakai S, Samukawa Y. Efficacy and safety of luseogliflozin as monotherapy in Japanese patients with type 2 diabetes mellitus: a randomized, double-blind, placebo-controlled, phase 3 study. Curr Med Res Opin. 2014 Jul;30(7):1245-55. doi:

10.1185/03007995.2014.912983. Epub 2014 Apr 29. PMID: 24708292.

3: Seino Y, Sasaki T, Fukatsu A, Ubukata M, Sakai S, Samukawa Y. Dose-finding study of luseogliflozin in Japanese patients with type 2 diabetes mellitus: a 12-week, randomized, double-blind, placebo-controlled, phase II study. Curr Med Res Opin. 2014 Jul;30(7):1231-44. doi: 10.1185/03007995.2014.909390. Epub 2014 Apr 15. Erratum in: Curr Med Res Opin. 2015 Dec;31(12):2345. PMID: 24673496.

4: Seino Y, Sasaki T, Fukatsu A, Sakai S, Samukawa Y. Efficacy and safety of luseogliflozin monotherapy in Japanese patients with type 2 diabetes mellitus: a 12-week, randomized, placebo-controlled, phase II study. Curr Med Res Opin. 2014 Jul;30(7):1219-30. doi: 10.1185/03007995.2014.901943. Epub 2014 Mar 19. PMID: 24597840.

**6. Tofogliflozin**

6.1. Search strategy

((tofogliflozin[Title]) AND (((((type 2 diabetes[Title]) OR (type 2 diabetes mellitus[Title])) OR (T2D[Title])) OR (T2DM[Title])) OR (type 2 diabetic patients[Title]))) AND ((((weight) OR (bodyweight)) OR (body mass index)) OR (BMI))

6.2. List of final included studies (n=3)

1: Terauchi Y, Tamura M, Senda M, Gunji R, Kaku K. Long-term safety and efficacy of tofogliflozin as add-on to insulin in patients with type 2 diabetes: Results from a 52-week, multicentre, randomized, double-blind, open-label extension, Phase 4 study in Japan (J-STEP/INS). Diabetes Obes Metab. 2018 May;20(5):1176-1185. doi: 10.1111/dom.13213. Epub 2018 Feb 11. PMID: 29316236; PMCID: PMC5947124.

2: Ikeda S, Takano Y, Cynshi O, Tanaka R, Christ AD, Boerlin V, Beyer U, Beck A, Ciorciaro C, Meyer M, Kadowaki T. A novel and selective sodium-glucose cotransporter-2 inhibitor, tofogliflozin, improves glycaemic control and lowers body weight in patients with type 2 diabetes mellitus. Diabetes Obes Metab. 2015 Oct;17(10):984-93. doi: 10.1111/dom.12538. Epub 2015 Aug 20. PMID: 26179482.

3: Kaku K, Watada H, Iwamoto Y, Utsunomiya K, Terauchi Y, Tobe K, Tanizawa Y, Araki E, Ueda M, Suganami H, Watanabe D; Tofogliflozin 003 Study Group. Efficacy and safety of monotherapy with the novel sodium/glucose cotransporter-2 inhibitor tofogliflozin in Japanese patients with type 2 diabetes mellitus: a combined Phase 2 and 3 randomized, placebo-controlled, double-blind, parallel-group comparative study. Cardiovasc Diabetol. 2014 Mar 28;13:65. doi: 10.1186/1475-2840-13-65. PMID: 24678906; PMCID: PMC4021346.

**Table S1. Studies identified for analysis of canagliflozin**

| Studies | Sources | Groups | Canagliflozin (mg/day) | Duration of treatment  (weeks) | Body weight (kg) | Number of people | Age  (years) | Duration of T2DM (years or months^*^) |
| --- | --- | --- | --- | --- | --- | --- | --- | --- |
| Yale JF  (2017) | Multinational | Canagliflozin | 100 | 52 | 80.7 (16.63) | 74 | 64.3 (8.49) | 9.7 (6.55) |
|  |  | Canagliflozin | 300 | 52 | 80.5 (18.97) | 74 | 65.8 (7.88) | 8.8 (6.24) |
|  |  | Control | - | 52 | 84.2 (18.97) | 69 | 64.3 (7.76) | 11.4 (7.15) |
| Kadowaki T  (2017) | Japan | Canagliflozin | 100 | 24 | 71.33 (15.94) | 70 | 58.4 (8.9) | 8.34 (7.74) |
|  |  | Control | - | 24 | 73.26 (12.91) | 68 | 56.0 (9.5) | 6.50 (3.89) |
| Inagaki N  (2016) | Japan | Canagliflozin | 100 | 16 | 69.95 ± 13.93 | 76 | 59.7 ± 9.4 | 15.18 ± 8.61 |
|  |  | Control | - | 16 | 69.68 ± 13.13 | 70 | 56.1 ± 10.9 | 12.34 ± 8.21 |
| Rodbard HW  (2016) | Multinational | Canagliflozin | 100 | 26 | 94.1 ± 21.8 | 107 | 57.4 ± 9.3 | 9.8 ± 5.4 |
|  |  | Control | - | 26 | 90.0 ± 19.3 | 106 | 57.5 ± 10.1 | 10.1 ± 5.9 |
| Rosenstock J  (2016) | Multinational | Canagliflozin | 100 | 26 | 88.3 ± 17.6 | 237 | 54.2 ± 9.6 | 2.9 ± 3.3 |
|  |  | Canagliflozin | 300 | 26 | 91.4 ± 21.4 | 237 | 55.4 ± 9.8 | 3.3 ± 3.9 |
|  |  | Control | - | 26 | 92.1 ± 20.1 | 237 | 55.2 ± 9.8 | 3.3 ± 4.5 |
| Fulcher G  (2016) | Multinational | Canagliflozin I | 100 | 18 | 91.5 | 103 | 62.4 (7.3) | 12.3 (6.2) |
|  |  | Canagliflozin I | 300 | 18 | 92.4 | 111 | 62.7 (7.7) | 13.2 (7.1) |
|  |  | Control I | - | 18 | 88.6 | 102 | 63.9 (8.3) | 12.5 (5.4) |
|  |  | Canagliflozin II | 100 | 18 | 109.2 | 35 | 60.7 (9.3) | 14.6 (5.8) |
|  |  | Canagliflozin II | 300 | 18 | 111.2 | 30 | 61.5 (7.4) | 15.3 (9.0) |
|  |  | Control II | - | 18 | 105.6 | 30 | 60.9 (7.3) | 14.7 (8.0) |
| Bode B  (2015) | Multinational | Canagliflozin | 100 | 104 | 88.4 ± 15.6 | 241 | - | 12.3 ± 7.8 |
|  |  | Canagliflozin | 300 | 104 | 88.8 ± 17.1 | 236 | - | 11.3 ± 7.2 |
|  |  | Control | - |  | 91.1 ± 17.5 | 237 | - | 11.4 ± 7.3 |
| Neal B  (2015) | Multinational | Canagliflozin | 100 | 52 | 94.4 (21.6) | 692 | 62.0 (32–83) | 16.4 (7.3) |
|  |  | Canagliflozin | 300 | 52 | 94.8 (21.3) | 690 | 63.0 (37–85) | 16.3 (7.4) |
|  |  | Control | - | 52 | 94.8 (22.3) | 690 | 63.0 (38–82) | 16.0 (7.8) |
| Ji L  (2015) | Multinational | Canagliflozin | 100 | 18 | 69.1 ± 11.8 | 223 | 56.5 ± 8.3 | 6.8 ± 4.5 |
|  |  | Canagliflozin | 300 | 18 | 69.6 ± 11.9 | 227 | 56.4 ± 9.2 | 6.9 ± 4.9 |
|  |  | Control | - | 18 | 68.6 ± 11.9 | 226 | 55.8 ± 9.4 | 6.4 ± 4.6 |
| Inagaki N  (2014) | Japan | Canagliflozin | 100 | 24 | 69.10 ± 14.48 | 90 | 58.4 ± 10.4 | 4.72 ± 4.59 |
|  |  | Control | - | 24 | 68.57 ± 15.15 | 93 | 58.2 ± 11.0 | 5.63 ± 5.76 |
| Yale JF  (2014) | Multinational | Canagliflozin | 100 | 52 | 90.5 (18.4) | 90 | 69.5 (8.2) | 15.6 (7.4) |
|  |  | Canagliflozin | 300 | 52 | 90.2 (18.1) | 89 | 67.9 (8.2) | 17.0 (7.8) |
|  |  | Control | - | 52 | 92.8 (17.4) | 90 | 68.2 (8.4) | 16.4 (10.1) |
| Sha S  (2014) | Germany | Canagliflozin | 300 | 12 | 92.3 (16.5) | 18 | 63.3 (4.0) | 8.6 (4.0) |
|  |  | Control | - | 12 | 94.7 (12.4) | 18 | 62.3 (6.8) | 8.4 (4.6) |
| Forst T  (2014) | Multinational | Canagliflozin | 100 | 26 | 94.2 ± 22.2 | 113 | 56.7 ± 10.4 | 10.5 ± 6.6 |
|  |  | Canagliflozin | 300 | 26 | 94.4 ± 25.9 | 114 | 57.0 ± 10.2 | 11.0 ± 7.6 |
|  |  | Control | - | 26 | 93.8 ± 22.4 | 115 | 58.3 ± 9.6 | 10.1 ± 6.6 |
| Wilding JP  (2013) | Multinational | Canagliflozin | 100 | 52 | 93.8 ± 22.6 | 157 | 57.4 ± 10.5 | 9.0 ± 5.7 |
|  |  | Canagliflozin | 300 | 52 | 93.5 ± 22.0 | 156 | 56.1 ± 8.9 | 9.4 ± 6.4 |
|  |  | Control | - | 52 | 91.2 ± 22.6 | 156 | 56.8 ± 8.3 | 10.3 ± 6.7 |
| Inagaki N  (2013) | Japan | Canagliflozin | 100 | 12 | 68.61 ± 14.86 | 74 | 57.7 ± 10.5 | - |
|  |  | Canagliflozin | 300 | 12 | 71.30 ± 12.19 | 75 | 57.1 ± 10.1 | - |
|  |  | Control | - | 12 | 72.56 ± 15.36 | 75 | 57.7 ± 11.0 | - |
| Stenlöf K  (2013) | Multinational | Canagliflozin | 100 | 26 | 85.8 (21.4) | 195 | 55.1 (10.8) | 4.5 (4.4) |
|  |  | Canagliflozin | 300 | 26 | 86.9 (20.5) | 197 | 55.3 (10.2) | 4.3 (4.7) |
|  |  | Control | - | 26 | 87.6 (19.5) | 192 | 55.7 (10.9) | 4.2 (4.1) |
| Rosenstock J  (2012) | Multinational | Canagliflozin | 100 | 12 | 87.7 ± 15.5 | 64 | 51.7 ± 8.0 | 6.1 ± 4.7 |
|  |  | Canagliflozin | 300 | 12 | 87.3 ± 15.9 | 64 | 52.3 ± 6.9 | 5.9 ± 5.2 |
|  |  | Control | - | 12 | 85.9 ± 19.5 | 64 | 53.3 ± 7.8 | 6.4 ± 5.0 |
| Devineni D  (2012) | USA | Canagliflozin | 100 | 4 | 107.8 (23.34) | 10 | 50.5 (9.58) | - |
|  |  | Canagliflozin | 300 | 4 | 94.1 (16.17) | 10 | 42.7 (6.24) | - |
|  |  | Control | - | 4 | 95.1 (13.96) | 9 | 52.8 (8.18) | - |

**Table S2. Studies identified for analysis of empagliflozin**

| Studies | Sources | Groups | Empagliflozin (mg/day) | Duration of treatment  (weeks) | Body weight (kg) | Number of people | Age  (years) | Duration of T2DM (years or months^*^) |
| --- | --- | --- | --- | --- | --- | --- | --- | --- |
| Kahl S  (2020) | Germany | Empagliflozin | 25 | 24 | 94.7 | 42 | 62.7 ± 7.0 | 36 ± 27^*^ |
|  |  | Control | - | 24 | 98.3 | 42 | 61.5±10.0 | 40 ± 27^*^ |
| Hattori S  (2018) | Japan | Empagliflozin | 10 | 12 | 83.55 ± 17.9 | 58 | - | - |
|  |  | Control | - | 12 | 80.7 ± 10.1 | 51 | - | - |
| Søfteland E  (2017) | Multinational | Empagliflozin | 10 | 24 | 88.4 (20.8) | 109 | 54.3 (9.6) | - |
|  |  | Empagliflozin | 25 | 24 | 84.4 (19.2) | 110 | 55.4 (9.9) | - |
|  |  | Control | - |  | 82.3 (19.8) | 108 | 55.9 (9.7) | - |
| Hadjadj S  (2016) | Multinational | Empagliflozin I | 10 | 24 | 82.3 ± 19.2 | 161 | 52.2 ± 11.7 | - |
|  |  | Empagliflozin I | 25 | 24 | 82.9 ± 18.7 | 165 | 51.0 ± 10.7 | - |
|  |  | Control I | - | 24 | 82.7 ± 21.2 | 166 | 53.4 ± 10.9 | - |
|  |  | Empagliflozin II | 10 | 24 | 83.0 ± 19.1 | 165 | 52.3 ± 11.3 | - |
|  |  | Empagliflozin II | 25 | 24 | 83.8 ± 19.8 | 167 | 53.6 ± 10.7 | - |
|  |  | Control II | - | 24 | 83.7 ± 20.1 | 162 | 51.6 ± 10.8 | - |
| Roden M  (2015) | Multinational | Empagliflozin | 10 | 76 | 78.4 (18.7) | 224 | 56.2 (11.6) | - |
|  |  | Empagliflozin | 25 | 76 | 77.8 (18.0) | 224 | 53.8 (11.6) | - |
|  |  | Control | - | 76 | 78.2 (19.9) | 228 | 54.9 (10.9) | - |
| Rosenstock J (2015) | Multinational | Empagliflozin | 10 | 78 | 91.6 ± 1.5 | 164 | 58.8 ± 9.9 | - |
|  |  | Empagliflozin | 25 | 78 | 94.7 ± 1.7 | 144 | 58.8 ± 9.9 | - |
|  |  | Control | - | 78 | 90.5 ± 1.7 | 160 | 58.8 ± 9.9 | - |
| Merker L  (2015) | Multinational | Empagliflozin | 10 | 76 | 81.6 (18.5) | 197 | 55.5 (9.9) | - |
|  |  | Empagliflozin | 25 | 76 | 82.2 (19.3) | 185 | 55.6 (10.2) | - |
|  |  | Control | - | 76 | 79.7 (18.6) | 158 | 56.0 (9.7) | - |
| DeFronzo RA (2015) | Multinational | Empagliflozin | 10 | 52 | 86.6 (19.0) | 135 | 56.2 (10.3) | - |
|  |  | Empagliflozin | 25 | 52 | 85.5 (20.4) | 134 | 57.1 (10.2) | - |
|  |  | Control | - | 52 | 85.0 (18.3) | 128 | 56.2 (10.0) | - |
| Kadowaki T (2014) | Japan | Empagliflozin | 10 | 12 | 68.1 ± 14.6 | 109 | 57.9 ± 9.4 | - |
|  |  | Empagliflozin | 25 | 12 | 68.3 ± 14.1 | 109 | 57.2 ± 9.7 | - |
|  |  | Control | - |  | 69.0 ± 12.2 | 109 | 58.7 ± 8.7 | - |
| Häring HU  (2014) | Multinational | Empagliflozin | 10 | 24 | 81.6 (18.5) | 217 | 55.5 (9.9) | - |
|  |  | Empagliflozin | 25 | 24 | 82.2 (19.3) | 213 | 55.6 (10.2) | - |
|  |  | Control | - | 24 | 79.7 (18.6) | 207 | 56.0 (9.7) | - |
| Ferrannini E (2013) | Multinational | Empagliflozin | 10 | 78 | 89.6 ± 15.0 | 166 | 60 (33-77 | - |
|  |  | Empagliflozin | 25 | 78 | 89.5 ± 16.2 | 166 | 60 (37-79) | - |
|  |  | Control | - | 78 | 85.8 ± 15.6 | 56 | 58 (35-73 | - |
| Kovacs CS  (2014) | Multinational | Empagliflozin | 10 | 24 | 78.0 ± 19.1 | 165 | 54.7 (9.9) | - |
|  |  | Empagliflozin | 25 | 24 | 78.9 ± 19.9 | 168 | 54.2 (8.9) | - |
|  |  | Control | - | 24 | 78.1 ± 20.1 | 165 | 54.6 (10.5) | - |
| Rosenstock J (2013) | Multinational | Empagliflozin | 10 | 12 | 87.9 ± 14.4 | 71 | 59 ± 9.0 | - |
|  |  | Empagliflozin | 25 | 12 | 90.5 ± 16.9 | 70 | 59 ± 8.1 | - |
|  |  | Control | - | 12 | 87.7 ± 15.7 | 71 | 60 ± 8.5 | - |
| Häring HU  (2013) | Multinational | Empagliflozin | 10 | 24 | 77.1 (18.3) | 225 | 57.0 (9.2) | - |
|  |  | Empagliflozin | 25 | 24 | 77.5 (18.8) | 216 | 57.4 (9.3) | - |
|  |  | Control | - | 24 | 76.2 (16.9) | 225 | 56.9 (9.2) | - |
| Ferrannini E (2013) | Multinational | Empagliflozin | 10 | 12 | 76.8 (45.5–118.0) | 81 | 58.0 (30–76) | - |
|  |  | Empagliflozin | 25 | 12 | 81.2 (49.1–130.0) | 82 | 57.0 (30–79) | - |
|  |  | Control | - | 12 | 82.2 (49.0–152.3) | 81 | 58.0 (28–80) | - |

**Table S3. Studies identified for analysis of ertugliflozin**

| Studies | Sources | Groups | Ertugliflozin (mg/day) | Duration of treatment  (weeks) | Body weight (kg) | Number of people | Age  (years) | Duration of T2DM (years or months^*^) |
| --- | --- | --- | --- | --- | --- | --- | --- | --- |
| Ji L  (2019) | Multinational | Ertugliflozin | 5 | 26 | 71.4 (11.1) | 170 | 56.1 (9.0) | 7.0 (5.0) |
|  |  | Ertugliflozin | 15 | 26 | 69.5 (10.9) | 169 | 56.3 (9.3) | 7.5 (5.1) |
|  |  | Control | - | 26 | 70.1 (12.4) | 167 | 56.9 (9.0) | 6.4 (5.1) |
| Gallo S  (2019) | Multinational | Ertugliflozin | 5 | 26 | 84.8 ± 17.2 | 207 | 56.6 ± 8.1 | 7.9 ± 6.1 |
|  |  | Ertugliflozin | 15 | 26 | 85.3 ± 16.5 | 205 | 56.9 ± 9.4 | 8.1 ± 5.5 |
|  |  | Control | - | 26 | 84.5 ± 17.1 | 209 | 56.5 ± 8.7 | 8.0 ± 6.3 |
| Aronson R  (2018) | Multinational | Ertugliflozin | 5 | 26 | 94.0 (25.4) | 156 | 56.8 (11.4) | 5.11 (5.09) |
|  |  | Ertugliflozin | 15 | 26 | 90.6 (18.3) | 152 | 56.2 (10.8) | 5.22 (5.55) |
|  |  | Control | - | 26 | 94.2 (25.2) | 153 | 56.1 (10.9) | 4.63 (4.52) |
| Pratley RE  (2018) | Multinational | Ertugliflozin | 5 | 26 | 89.5 (20.8) | 243 | 55.2 (10.4) | 7.0 (5.6) |
|  |  | Ertugliflozin | 15 | 26 | 87.5 (20.5) | 244 | 55.1 (9.8) | 6.9 (5.2) |
|  |  | Control | - | 26 | 89.8 (23.5) | 247 | 54.8 (10.7) | 6.2 (5.2) |
| Dagogo-Jack S  (2018) | Multinational | Ertugliflozin | 5 | 26 | 87.6 (18.6) | 156 | 59.2 (9.3) | 9.9 (6.1) |
|  |  | Ertugliflozin | 15 | 26 | 86.6 (19.5) | 153 | 59.7 (8.6) | 9.2 (5.3) |
|  |  | Control | - | 26 | 86.4 (20.8) | 153 | 58.3 (9.2) | 9.4 (5.6) |
| Amin NB  (2015) | Multinational | Ertugliflozin | 5 | 12 | - | 55 | 54.7 ± 7.7 | 6.7 |
|  |  | Control | - | 12 | - | 54 | 54 ± 8.1 | 6.4 |
| Grunberger G  (2018) | Multinational | Ertugliflozin | 5 | 26 | 89.4 (22.5) | 158 | 66.7 (8.3) | 14.9 (9.0) |
|  |  | Ertugliflozin | 15 | 26 | 85.8 (17.4) | 155 | 67.5 (8.5) | 14.5 (8.5) |
|  |  | Control | - | 26 | 90.4 (18.9) | 154 | 67.5 (8.9) | 13.1 (8.1) |

**Table S4. Studies identified for analysis of ipragliflozin**

| Studies | Sources | Groups | Ipragliflozin (mg/day) | Duration of treatment  (weeks) | Body weight (kg) | Number of people | Age  (years) | Duration of T2DM (years or months^*^) |
| --- | --- | --- | --- | --- | --- | --- | --- | --- |
| Inoue H  (2019) | Japan | Ipragliflozin | 50 | 24 | 73.6 ± 14.7 | 24 | 60.5 ± 9.8 | 15.9 ± 7.7 |
|  |  | Control | - | 24 | 74.6 ± 13.3 | 24 | 60.8 ± 12.1 | 19.1 ± 10.7 |
| Shestakova MV(2018) | Russian | Ipragliflozin | 50 | 24 | 92.41 ± 14.53 | 38 | 58.7 ± 9.9 | 73.3 ± 71.1^*^ |
|  |  | Control | - | 24 | 96.26 ± 14.40 | 14 | 54.4 ± 10.9 | 69.7 ± 63.4^*^ |
| Han KA  (2018) | Korean | Ipragliflozin | 50 | 24 | 67.50 (12.50) | 73 | 57.62 (8.26) | 139.41 (70.73)^*^ |
|  |  | Control | - | 24 | 67.90 (10.98) | 66 | 57.44 (7.88) | 135.98 (79.55)^*^ |
| Ishihara H  (2016) | Japan | Ipragliflozin | 50 | 16 | 69.05 ± 11.61 | 168 | 58.7 ±11.1 | 151.1 ± 93.5^*^ |
|  |  | Control | - | 16 | 70.32 ± 12.17 | 87 | 59.2 ± 9.3 | 171.4 ± 102.5^*^ |
| Kashiwagi A  (2015) | Japan | Ipragliflozin | 50 | 24 | 69.16 ± 11.57 | 118 | 63.9 ± 6.59 | 114.3 ± 92.26^*^ |
|  |  | Control | - | 24 | 66.70 ± 10.94 | 46 | 65.7 ± 6.93 | 113.0 ± 99.77^*^ |
| Kashiwagi A  (2015) | Japan | Ipragliflozin | 50 | 24 | 68.52 ± 13.86 | 112 | 56.2 (10.67) | 89.9 (68.10)^*^ |
|  |  | Control | - | 24 | 67.51 ± 11.36 | 56 | 57.7 (9.24) | 96.6 (61.93)^*^ |
| Kadokura T  (2014) | Japan | Ipragliflozin | 50 | 2 | 68.44 ± 13.58 | 9 | 59.3 ± 9.64 | - |
|  |  | Ipragliflozin | 100 | 2 | 75.07 ± 13.89 | 9 | 57.0 ± 13.19 | - |
|  |  | Control | - | 2 | 66.72 ± 7.72 | 10 | 60.0 ± 7.72 | - |
| Fonseca VA  (2013) | Multinational | Ipragliflozin | 50 | 12 | 90.7 ± 20.8 | 67 | 52.6 ± 10.7 | 4.61 ± 4.65 |
|  |  | Control | - | 12 | 81.8 ± 17.6 | 69 | 53.4 ± 9.7 | 4.64 ± 5.93 |
| Wilding JP  (2013) | Multinational | Ipragliflozin | 50 | 12 | 86.7 ± 13.7 | 68 | 58.6 ± 7.6 | 6.0 ± 5.3 |
|  |  | Control | - | 12 | 89.0 ± 14.5 | 66 | 57.3 ± 8.6 | 5.7 ± 3.2 |
| Schwartz SL  (2011) | Multinational | Ipragliflozin | 50 | 5 | 85.1 ±12.7 | 12 | 57.7 ± 9.1 | - |
|  |  | Ipragliflozin | 100 | 5 | 93.8 ±13.1 | 12 | 57.3 ± 9.5 | - |
|  |  | Control | - | 5 | 89.3 ± 13.5 | 13 | 53.3 ± 11.9 | - |

**Table S5. Studies identified for analysis of luseogliflozin**

| Studies | Sources | Groups | Luseogliflozin (mg/day) | Duration of treatment  (weeks) | Body weight (kg) | Number of people | Age  (years) | Duration of T2DM (years or months^*^) |
| --- | --- | --- | --- | --- | --- | --- | --- | --- |
| Seino Y  (2018) | Japan | Luseogliflozin | 2.5 | 16 | 68.10 (11.32) | 159 | 57.4 (10.3) | 11.7 (7.6) |
|  |  | Control | - | 16 | 69.13 (12.16) | 74 | 57.1 (10.9) | 12.1 (6.8) |
| Seino Y  (2014) | Japan | Luseogliflozin | 2.5 | 24 | 70.19 (13.65) | 79 | 58.9 (10.1) | 6.5 (5.9) |
|  |  | Control | - | 24 | 66.67 (11.23) | 79 | 59.6 (9.3) | 6.1 (5.4) |
| Seino Y  (2014) | Japan | Luseogliflozin | 2.5 | 12 | 66.67 (11.25) | 56 | 57.4 (9.3) | 4.6 (4.4) |
|  |  | Luseogliflozin | 5 | 12 | 72.56 (13.94) | 54 | 57.3 (11.4) | 4.5 (4.2) |
|  |  | Control | - | 12 | 67.32 (13.14) | 57 | 57.1 (10.0) | 5.1 (4.6) |
| Seino Y  (2014) | Japan | Luseogliflozin | 2.5 | 12 | 65.5 (12.2) | 61 | 58.3 (9.4) | 6.15 (6.50) |
|  |  | Luseogliflozin | 5 | 12 | 66.3 (12.4) | 61 | 56.8 (9.3) | 5.77 (5.55) |
|  |  | Control | - | 12 | 68.3 (13.4) | 54 | 57.6 (11.0) | 7.30 (6.43) |

**Table S6. Studies identified for analysis of tofogliflozin**

| Studies | Sources | Groups | Tofogliflozin (mg/day) | Duration of treatment  (weeks) | Body weight (kg) | Number of people | Age  (years) | Duration of T2DM (years or months^*^) |
| --- | --- | --- | --- | --- | --- | --- | --- | --- |
| Terauchi Y  (2018) | Japan | Tofogliflozin | 20 | 16 | 68.84 ± 13.24 | 140 | 59.1 ± 10.9 | 15.06 ± 9.39 |
|  |  | Control | - | 16 | 72.24 ± 11.12 | 70 | 56.4 ± 10.0 | 12.39 ± 7.34 |
| Ikeda S  (2015) | Japan | Tofogliflozin | 20 | 12 | 84.91(17.305) | 64 | 56.3(9.79) | 5.21(3.934) |
|  |  | Tofogliflozin | 40 | 12 | 81.68(18.692) | 67 | 57.5(9.31) | 6.44(5.811) |
|  |  | Control | - | 12 | 83.73(19.201) | 66 | 53.9(11.12) | 5.98(5.287) |
| Kaku K  (2014) | Japan | Tofogliflozin | 20 | 24 | 68.06 (15.82) | 58 | 56.6 (10.2) | 6.4 (5.1) |
|  |  | Tofogliflozin | 40 | 24 | 68.72 (11.91) | 58 | 57.0 (9.1) | 6.7 (5.5) |
|  |  | Control | - | 24 | 71.20 (12.64) | 56 | 56.8 (9.9) | 6.0 (6.1) |

**Table S7. Parameter estimates of final models and boostrop (n =1000)**

| Model | Parameter | Estimate | Boostrap  Median 95% CI | | Model | Parameter | Estimate | Boostrap  Median 95% CI | | |
| --- | --- | --- | --- | --- | --- | --- | --- | --- | --- | --- |
| (A) | E_max_, % | -3.72 | -3.72 | [-3.72, -3.72] | (B) | E_max_, % | -5.59 | | -5.59 | [-5.59, -2.97] |
|  | ET_50_, week | 3.35 | 1.00 | [1.00, 6.81] |  | ET_50_, week | 16.8 | | 15.6 | [4.18, 28.4] |
| (C) | E_max_, % | -2.84 | -2.84 | [-2.84, -2.45] | (D) | E_max_, % | -3.43 | | -3.43 | [-3.43, -1.62] |
|  | ET_50_, week | 3.42 | 3.42 | [1.32, 5.49] |  | ET_50_, week | 3.09 | | 2.74 | [0.25, 6.31] |
| (E) | E_max_, % | -3.04 | -3.04 | [-3.04, -3.04] | (F) | E_max_, % | -2.45 | | -2.45 | [-2.45, -2.45] |
|  | ET_50_, week | 4.38 | 4.31 | [3.73, 5.19] |  | ET_50_, week | 3.16 | | 3.09 | [2.04, 4.01] |

(A), canagliflozin; (B), empagliflozin; (C), ertugliflozin; (D), ipragliflozin; (E), luseogliflozin; (F), tofogliflozin; E_max_, the maximal effects; ET_50_, the treatment duration to reach half of the the maximal effects. CI: confidence interval.

**
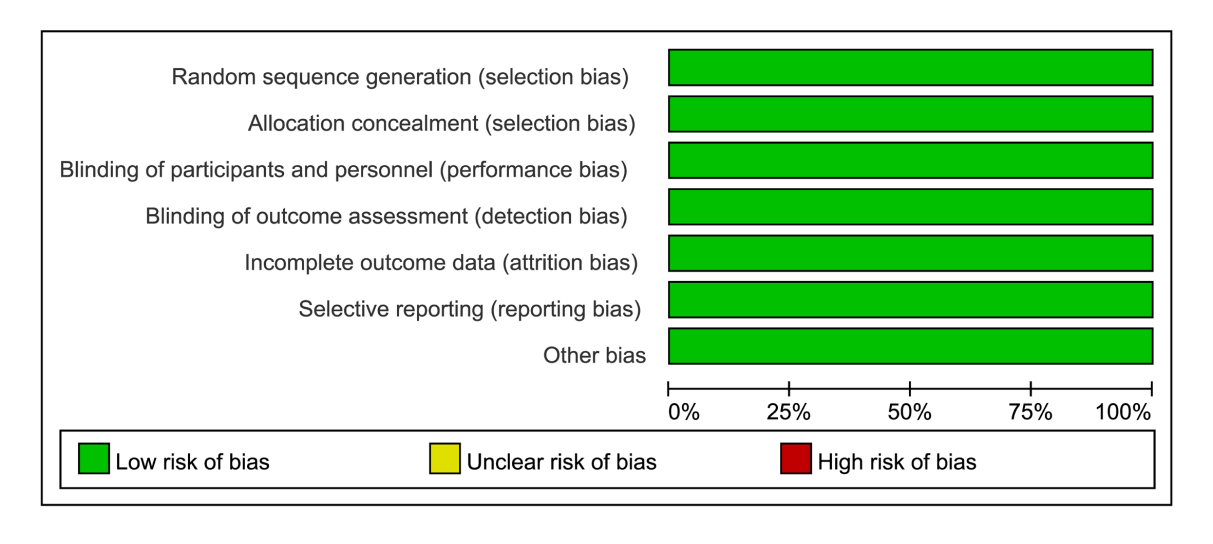
**

**Figure S1. The risk of bias of canagliflozin**

**
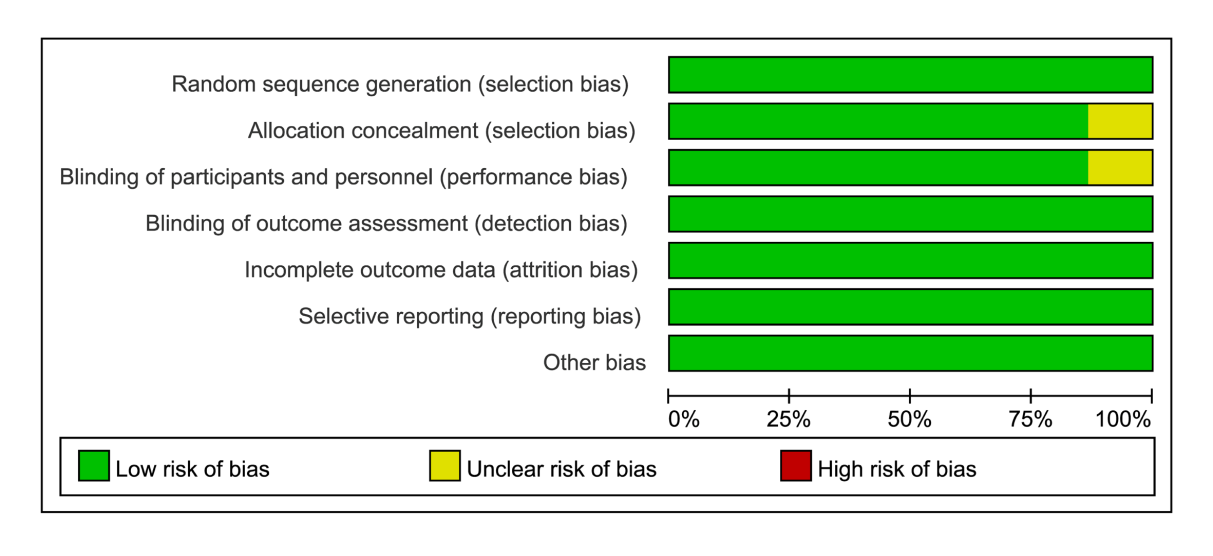
**

**Figure S2. The risk of bias of empagliflozin**

**
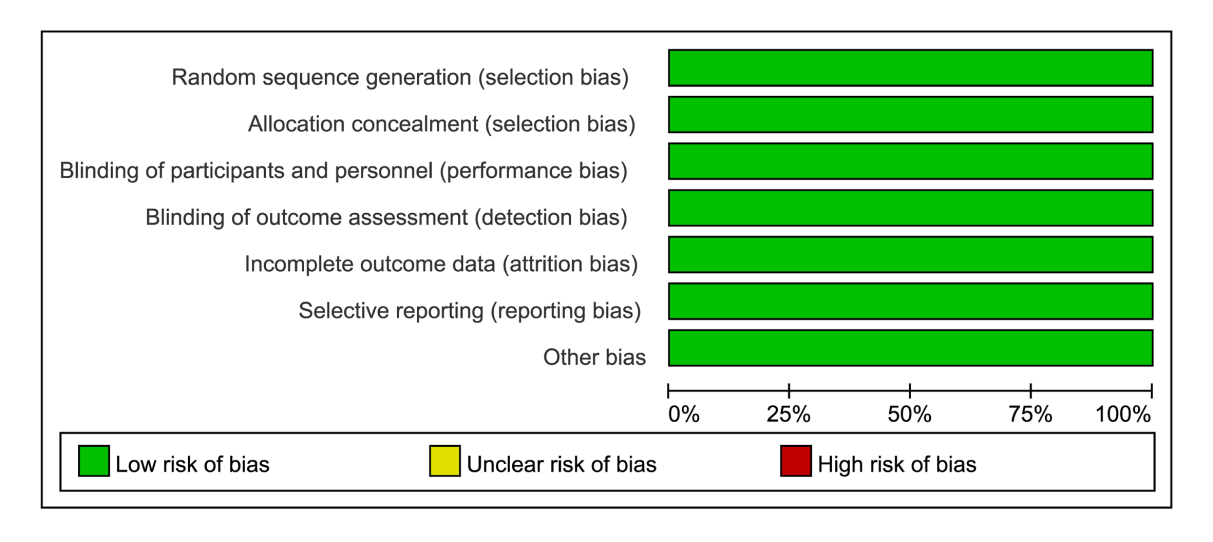
**

**Figure S3. The risk of bias of ertugliflozin**

**
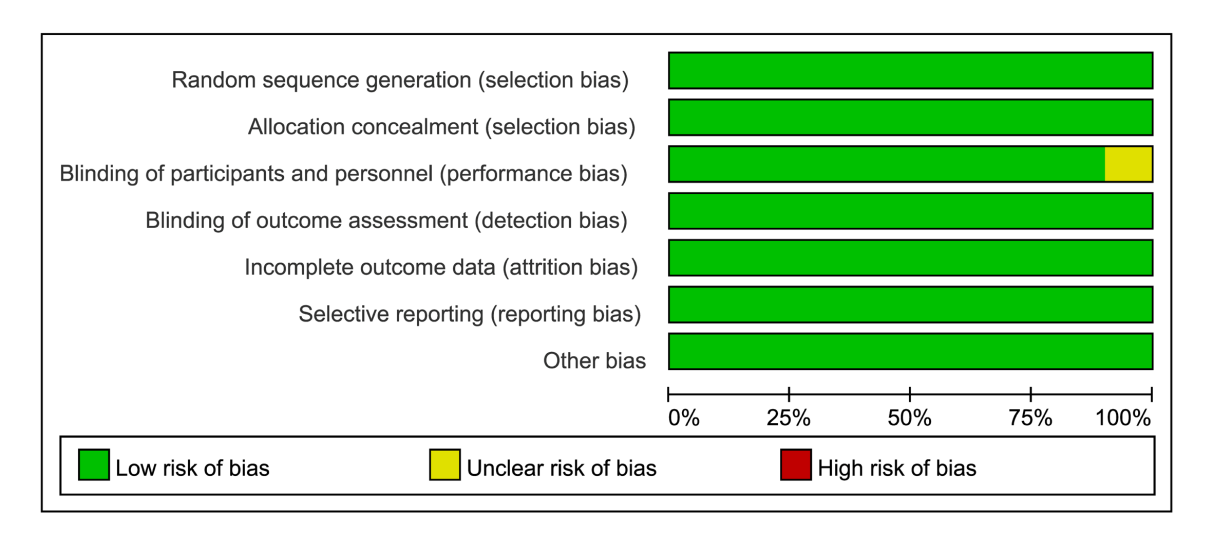
**

**Figure S4. The risk of bias of ipragliflozin**

**
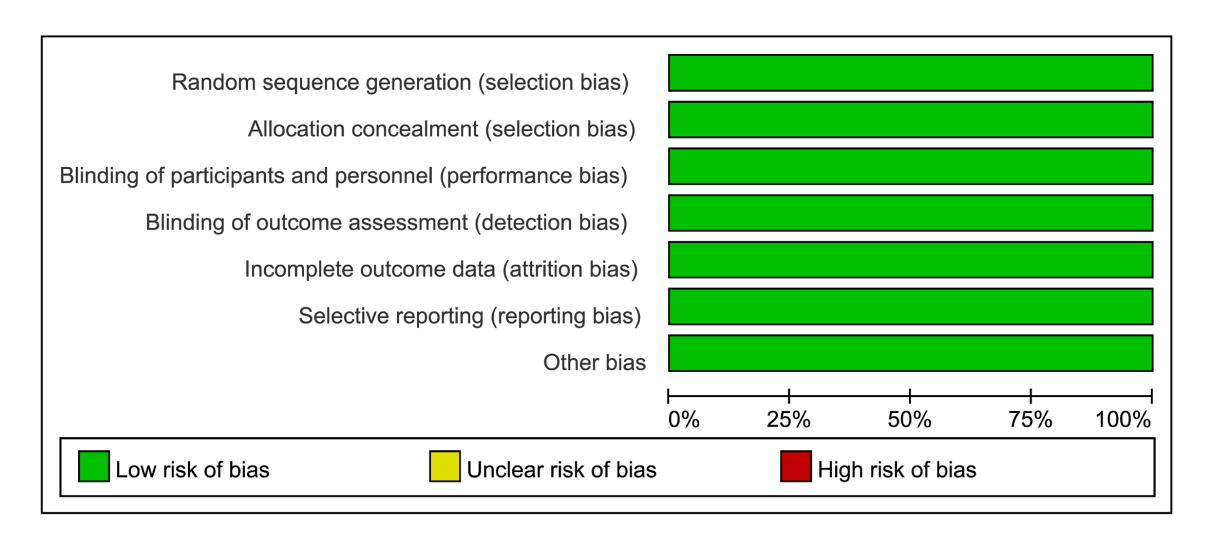
**

**Figure S5. The risk of bias of luseogliflozin**

**
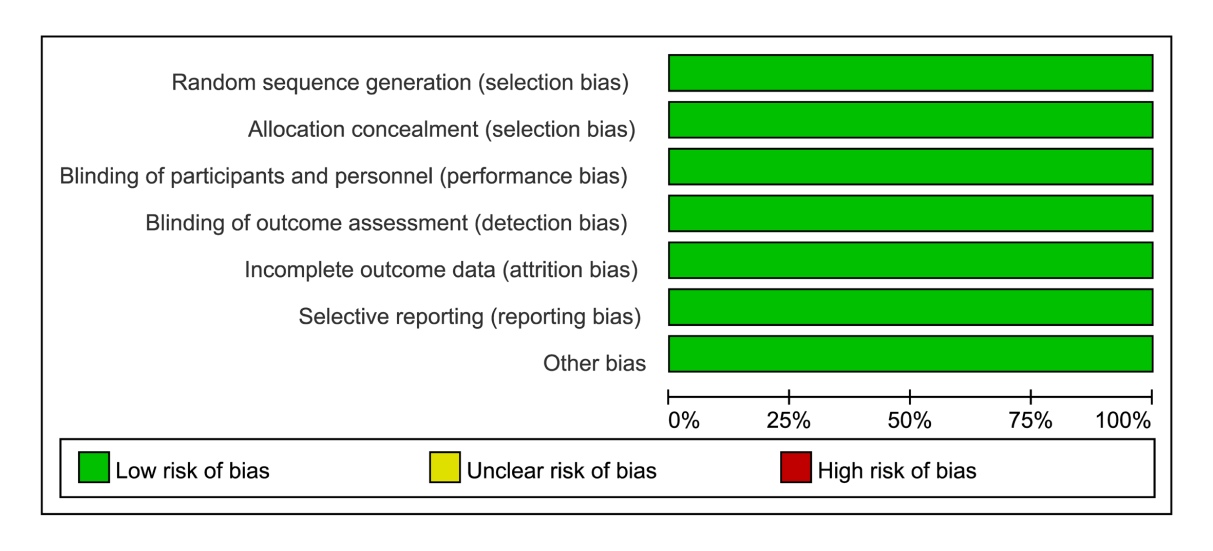
**

**Figure S6. The risk of bias of tofogliflozin**
